# Identifying the Therapeutic Significance of Mesenchymal Stem Cells

**DOI:** 10.3390/cells9051145

**Published:** 2020-05-06

**Authors:** Vineet Kumar Mishra, Hui-Hsuan Shih, Farzana Parveen, David Lenzen, Etsuro Ito, Te-Fu Chan, Liang-Yin Ke

**Affiliations:** 1 Department of Medical Laboratory Science and Biotechnology, College of Health Sciences, Kaohsiung Medical University, Kaohsiung 807378, Taiwan; vineetkmishra.jh@gmail.com (V.K.M.); fparveen.jh@gmail.com (F.P.); dav.lenzen@gmail.com (D.L.); 2 Center for Lipid Biosciences, Kaohsiung Medical University Hospital & Lipid Science and Aging Research Center, Kaohsiung Medical University, Kaohsiung 807377, Taiwan; 3 Division of Chest Medicine, Department of International Medicine, MacKay Memorial Hospital, Taipei 104217, Taiwan; huihsuan.shih@gmail.com; 4 Graduate Institute of Medicine, College of Medicine & Drug Development and Value Creation Research Center, Kaohsiung Medical University, Kaohsiung 807378, Taiwan; eito@waseda.jp; 5 Department of Biology, Waseda University, Tokyo 162-8480, Japan; 6 Waseda Research Institute for Science and Engineering, Waseda University, Tokyo 162-8480, Japan; 7 Department of Obstetrics and Gynecology, Kaohsiung Medical University Hospital, Kaohsiung 807377, Taiwan

**Keywords:** mesenchymal stem cell (MSC), microenvironment, immunosuppression, immunomodulation, adipogenesis, type 2 diabetes

## Abstract

The pleiotropic behavior of mesenchymal stem cells (MSCs) has gained global attention due to their immense potential for immunosuppression and their therapeutic role in immune disorders. MSCs migrate towards inflamed microenvironments, produce anti-inflammatory cytokines and conceal themselves from the innate immune system. These signatures are the reason for the uprising in the sciences of cellular therapy in the last decades. Irrespective of their therapeutic role in immune disorders, some factors limit beneficial effects such as inconsistency of cell characteristics, erratic protocols, deviating dosages, and diverse transfusion patterns. Conclusive protocols for cell culture, differentiation, expansion, and cryopreservation of MSCs are of the utmost importance for a better understanding of MSCs in therapeutic applications. In this review, we address the immunomodulatory properties and immunosuppressive actions of MSCs. Also, we sum up the results of the enhancement, utilization, and therapeutic responses of MSCs in treating inflammatory diseases, metabolic disorders and diabetes.

## 1. Introduction

MSCs are Mesenchymal Stem Cells (MSCs), which can be defined as non-hematopoietic multipotent stem cells with the ability to differentiate into mesodermal lineage (adipocytes, osteocytes and chondrocytes), ectodermal lineage (neurocytes) and endodermal lineage (hepatocytes) [1,2]. In 1966, Friedenstein and his team discovered the multipotent behavior of MSCs for the first time [3]. After many years, the term MSCs was coined by Caplan et al. in 1991 [4]. Since then, MSCs have become a well-known and outstanding cell source alluring for clinical applications. They have an excellent capacity for self-renewal in vitro, lasting for more than four months [5].

Earlier, there was a debate among scientists about the stemness and nomenclature of MSCs. Some articles preferred mesenchymal “stromal” cells instead of stem cells [6]. Some researchers attempted to change the name of MSCs to medicinal signaling cells because of their secretory role in the locations of diseases, injuries, and inflammations [7,8]. However, later reports showed that prostaglandin E2 (PGE2) secreted by MSCs is responsible for maintaining the self-renewal ability and PGE2 is also involved in the immunomodulation of MSCs, creating a cascade of events, which proves the stemness of MSCs [9]. Hence, the term Mesenchymal Stem Cells is justified.

There are various sources of isolation of MSCs such as adipose tissue, bone marrow, peripheral blood and neonatal tissues (umbilical cord, placenta, amniotic fluid, and amniotic membrane) [2,10,11,12]. The source of isolation of MSCs greatly affects the yield, the expressed variety of surface markers and cytokine profile [13,14,15]. However, the basic identification markers of MSCs are CD73 (cluster of differentiation 73), CD105, CD90 but they should be CD34-, CD14-, CD45-, CD11b-, CD19- and lack HLAII (Human Leukocyte Antigen complex 2) [16]. Besides that, MSCs must express transcription factors such as octamer-binding transcription factor 4 (OCT-4) and homeobox protein NANOG [17].

Currently, there is a lack of standardized methods for the isolation and culture of MSCs [18]. Comparative studies are challenging due to MSCs showing different features depending on their source and microenvironment from which they are isolated [19,20,21]. Several researchers focused on optimizing the isolation of MSCs. Talwadekar et al. found out that the clonogenicity and function of placenta MSCs (P-MSCs) were superior to cord MSCs (C-MSCs) [22]. Similarly, Nagaishi et al. showed that MSCs from umbilical cord significantly improve diabetic abnormalities and diabetic nephropathy in comparison to the bone marrow-derived MSCs [23]. Therefore, the source of MSC isolation is critical in context with its potential and efficacy towards its properties [2,23]. 

In this review, we aim to discuss the immunomodulatory properties and immunosuppressive actions of MSCs. Besides that, we sum up the results of the enhancement, utilization, and therapeutic responses of MSCs in treating human diseases, and particularly their potential towards diabetes and adipose tissue dysfunction.

## 2. The Immunological Functions of Mesenchymal Stem Cells

The primary functions of MSCs are: (1) immunomodulation, (2) autocrine and paracrine activities, and (3) evasion of innate immunity. Below, we discuss every aspect in detail.

### 2.1. Immunomodulation

**Figure 1:**
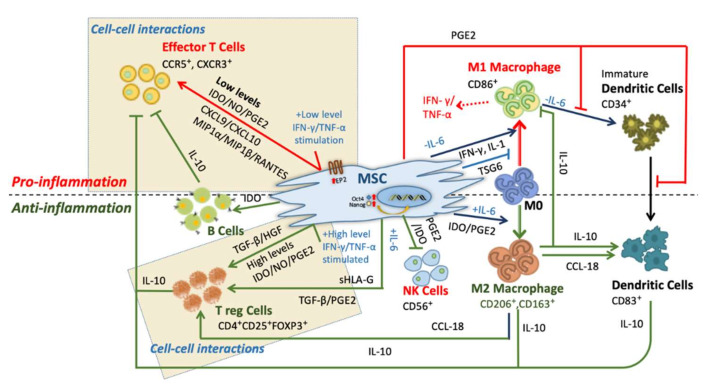
The central role of Mesenchymal Stem Cells (MSC) in immune responses. The above figure distinguishes the response and interaction of MSCs in pro-inflammatory and anti-inflammatory conditions on immune cells. These effects demonstrate cell-to-cell contact-mediated immunosuppression of B and T cell proliferation, induction and transforming growth factor-β (TGF-β)/hepatocyte growth factor (HGF) mediated regulation of regulatory T cells. Also, it shows the capacity of immunomodulation of MSCs by inhibiting the natural killer (NK) cells, dendritic cells (DCs) at various maturation stages as well as macrophage polarization dependency on the microenvironment.

The mode of immunomodulation is mediated by cell-cell interactions, cytokines and soluble factors. Note that, depending on the levels of stimulation, MSCs play either pro-inflammatory or anti-inflammatory roles in its microenvironment (Figure 1) [13,15,24,25].

#### 2.1.1. Pro-Inflammation

At low levels of interferon-gamma (IFN-γ) and tumor necrosis factor-α (TNF-α), MSCs show the pro-inflammatory phenotype (Figure 1, red arrows). MSCs produce chemokines such as macrophage inflammatory protein-1α/β (MIP-1α/β), RANTES, chemokine (C-X-C motif) ligand 9 (CXCL9), and CXCL10 within its microenvironment to activate the T cells [26]. MSCs secreting PGE-2 severely hamper dendritic cell (DC) precursors during the process of differentiation and maturation [27,28]. In the absence of interleukin-6 (IL-6), MSCs promote proliferation and activation of M1 macrophage [29]. The transition from M0 to pro-inflammatory M1 macrophage is induced by secretion of interferon-γ (IFN-γ) and IL-1, along with surface protein expression of CD40 ligand (CD40L). Further, these M1 macrophages express IFN-γ and tumor necrosis factor-α (TNF-α) within the microenvironment along with the co-stimulatory surface molecules to respond to T cell activation [29,30]. In contrast, in an anti-inflammatory microenvironment, this phenomenon can be inhibited by TNF-stimulated gene 6 protein (TSG-6) [31]. The feedback mechanism studied under in-vitro conditions of pro-inflammatory cytokines make MSCs enhance the immune response by producing chemokines CXCL-9, CXCL-10, and CXCL-11. This leads to more neutrophils moving towards the site of inflammation where they act mainly by phagocytosis [26,32]. The point to be noticed here is that human and mice derived MSCs have these effects only when exposed to low or insufficient levels of pro-inflammatory cytokines such as IFN-γ and TNF-α.

#### 2.1.2. Anti-Inflammation

In the anti-inflammatory condition, MSCs suppress the immune response in inflammatory cytokine-rich microenvironments, such as wounds, infections, or organ transplantations. These immunosuppressive indications were observed in humans [33,34,35,36,37], baboons [37], and murine [38], where MSCs successfully inhibited T lymphocyte activation and proliferation (Figure 1, green arrows). This particular behavior of MSCs in the presence and absence of inflammatory molecules is called MSC polarization [29]. Under high levels of IFN-γ and TNF-α, MSCs produce cytokines such as transforming growth factor-β (TGF-β), hepatocyte growth factor (HGF) [39], and secrete soluble factors such as indoleamine 2,3-dioxygenase (IDO), PGE2 and nitric oxide (NO) [40]. These factors directly promote the activation of regulatory T cells (Tregs) (CD4^+^, CD25^+^, forkhead box P3 (FOXP3^+^)). Besides, in responding to IL-6 stimulation, MSCs secrete TGF-β and PGE2 again to induce Treg cell activation.

MSCs can also promote the activation of T-reg cells indirectly. Recently Heo et al. described the M2 macrophage stimulation and phenotype changes by the exosomes derived from AD-MSCs in an in- vitro study [41]. Furthermore, these M2 macrophages (MSC stimulated) express CCL-18 and activate Tregs cells [42]. The process of immunosuppression is also dependent on factors released by MSCs or in the microenvironment. MSCs trigger the expression of cyclooxygenase 2 (COX2) and IDO and further promote homeostatic response towards macrophage polarization [15,43]. In that response, M2 macrophage expresses CD206 and CD163 co-stimulatory molecules, along with enriching the microenvironment by IL-6 and IL-10 expression [44]. The excessive IL-10 production by DCs and M2 upon MSCs co-culture further boosts the immunosuppression by suppressing effector T cells [45,46]. 

MSCs trigger the proliferation, activation and immunoglobulin G (IgG) secretion of B cells by IDO [47,48,49]. These again inhibit other T-effector cells to support anti-inflammation (Figure 1) [29,44,50]. Human- and mouse-derived BM-MSCs release specific chemokine ligands such as C-C motif chemokine ligand 2 (CCL2), CCL3 and CCL12 which help them to work with monocytes and macrophages in tissue homeostasis and wound healing [51].

### 2.2. Autocrine and Paracrine Role of MSC Secreted Molecules

MSCs secrete biomolecules such as growth factors, cytokines and chemokines which help their biological activities in the autocrine or paracrine manner in consonance to the encompassing microenvironment [52,53].

**Figure 2:**
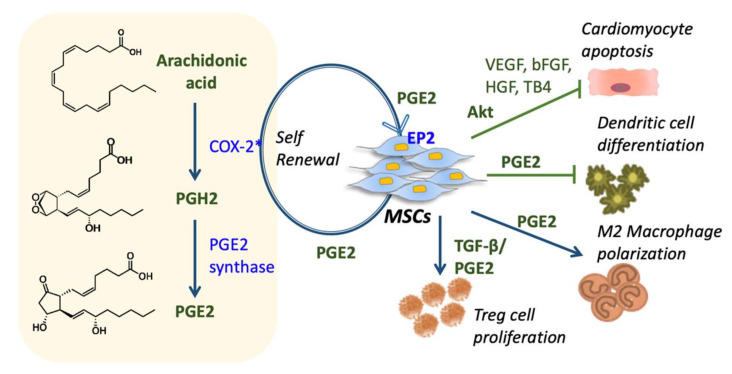
The autocrine and paracrine functions of MSC. The left side depicts the cyclooxygenase-2 (COX-2)/prostaglandin E2 (PGE2) axis for the maintenance of an autocrine/paracrine loop and COX-2 mediated PGE2 production in MSCs as a response to the surrounding microenvironment. The right side of the figure demonstrates the dominance of MSCs on immune cells (inhibiting cardiomyocyte apoptosis and DC differentiation, also promoting M2 macrophage polarization and T-reg cell proliferation) by producing several immunomodulatory factors and chemokines. Note: Figure represents both conditioned/modified and natural MSCs.

A typical autocrine manner of MSCs for maintaining self-renewal capacity is through PGE2 signaling [9] (Figure 2). PGE2 belongs to the prostaglandin family. It plays a role in lipid mediation and other physiological effects. For synthesizing PGE2, the conversion of arachidonic acid to prostaglandin H2 (PGH2) is regulated by COX-2, a prostaglandin-endoperoxidase synthase; and the isomerization of PGH2 to PGE2 is induced by PGE2 synthase [54]. COX-2 is the rate-limiting enzyme to mediate inflammatory cytokines, growth factors and tumor promoters [55,56]. MSCs secrete PGE2 to the extracellular environment by multidrug-resistant protein 4 (MRP4), later it binds to the PGE2 receptors on target cells [57]. PGE2 receptor 2 (EP2) augments cell proliferation and neovascularization by advancing the secretion of vascular endothelial growth factors (VEGF) [55,58,59]. By reviewing considerable amounts of in vitro and in vivo studies of immune disorders, we can say that COX-2 mediated PGE2 expression by MSCs are pivotal factors for the immunomodulatory ability of MSCs [60,61,62,63,64]. The loop between COX-2 and PGE2 maintains an axis that influences cell-cycle, cell-proliferation and cell viability by invigoration of one or more EP receptors [65].

MSCs are very competent in suppressing and tolerating the immune system via cell-cell contacts and soluble factors production. The migratory ability of MSCs toward inflammatory signals and damaged tissues makes them an excellent vector for therapeutics. Gnecchi et al. characterized MSCs and demonstrated the paracrine mechanism by genetically engineered protein kinase B (PKB, also known as Akt)-MSCs [66,67] (Figure 2). In this in vitro experiment, Akt-MSCs-derived conditioned medium collected from hypoxic Akt-MSCs was found to be efficiently protective to cardiomyocytes by inhibiting the apoptosis. The paracrine effect of MSCs became clearer when they found the effect of other valuable factors such as VEGF, bFGF (basic fibroblast growth factors), HGF and thymosin β-4 (TB4). 

The paracrine mechanism plays a major role in immunomodulation, where MSCs intensify their action in localized lymphocytes by the factors involving TGF-β, HGF, PGE2, human leukocyte antigen-G5 (HLAG-5), IL-6, CCL-2, CCL-5 and some other chemokines [68,69,70,71]. In the mammalian immune system, DCs are considered to be one of the most important antigen-presenting cells (APCs). However, due to the effects of MSCs, immature DC precursors (CD34^+^ precursors) ceased at the differentiation [72,73,74]. PGE2 from MSCs has been reported for its tolerogenic features, such as IL-10 release, IDO1 expression, also when merged with TNF-α, IL-1β, and IL6, it intensifies the immunogenicity with the co-stimulatory molecules [75,76,77]. In the vicinity of microenvironments treated with MSCs, mature DCs (CD83^+^ DCs with CD80 and CD86 costimulatory molecules) get obstructed in their efficiency for T cell activation [74,78]. Together, all these factors and chemokines generate tolerance against the immune cells and help MSCs to facilitate favorable therapeutic roles.

### 2.3. Escape Mechanism of MSCs From the Innate Immune System

MSCs escape from the immune system by the cell-cell interactions through the production of immune regulatory molecules such as IFN-γ, COX 2, PGE2 and IDO [26,79,80]. Among these factors, IFN-γ plays a crucial role in inducing IDO expression and tryptophan depletion for the escape mechanism [79]. In addition, HGF and TGF-β support MSCs to develop resistance against immune cells [81]. Meisel et al. provided comprehensive in vitro results, where the T cell proliferation was partly restored after adding monoclonal antibodies against TGF-β or HGF to show that both cytokines are participating in this interplay [82]. 

Previously, both autologous and allogeneic MSCs were killed by activated natural killer cells (NK cells) due to surface receptors such as MHC class I-related chain A (MIC-A), UL16-binding protein (ULBPs), poliovirus receptor and nectin-2 [83,84]. However, IFN-γ treated MSCs are less likely to be killed by NK cells [84]. MSCs inhibit their cytokines production, cytotoxicity, and cell proliferation through PGE2 and IDO [85,86]. In a nutshell, there are three large and extensive laps of the immune response: The first is antigen recognition and presentation, the second is T cell activation, proliferation and differentiation and the third is the effective stage. The escape mechanism of MSCs can be seen throughout all the three stages of immune responses. 

### 2.4. Complement System and MSCs

The complement system of a body is the first line of defense against pathogenic foreign invaders [87]. There are more than 30 proteins involved in the interplay of human complement system during an inflammatory response [88]. The three humanistic pathways by which the complement system activates are the classical, lectin and alternative pathway. The classical pathway involves complement component 1q (C1q) which is activated by antibodies. The lectin pathway becomes activated by carbohydrate moieties, and the alternative pathway is stimulated by hydrolysis of complement component 3 (C3). The ultimate result of this activation is to form a membrane attack complex (MAC) for the initiation of cell lysis [89]. 

#### 2.4.1. Complement System Attack on MSCs

It is well documented that during an infusion of MSCs to study clinical approaches, MSCs are prone to attract the complements in the blood and suffer damage caused by MACs. Injured MSCs are more vulnerable to further immune responses and also get compromised in their potent functionality [90]. Receptors present on monocytes such as CR3 (complement receptor 3) assist in phagocytosis of complement opsonized MSCs [91]. In some MSC-infusions in vivo experiments, a particular suppressive cell population has emerged along with M2 monocytes, observed to decrease the MSC population [15,92,93,94]. The interactions of MSCs with blood plasma require more extensive research to find the reason behind MSC depletion after infusion. Recently, Gavin et al. demonstrated the C3 mediated complement phagocytosis of MSCs by monocytes with markers CD14^+^CD16^-^ excluding the involvement of C5 complex [95]. Such examples may answer the question of depletion in the population of MSC after infusion.

#### 2.4.2. MSCs’ Ability to Counteract the Complement System

The MSC has surface receptors C3aR (C3a receptor) and C5aR (C5a receptor). During a surrounding inflammatory action in the presence of C3a and C5a, MSCs express them on their surface and bind to generate resistance against oxidative stress and apoptosis-inducing mechanisms [96]. Additionally, MSCs have surface expression of CD46, CD55 and CD59, which protect MSCs against the complement action and consequently prevent cell death [96,97]. Although, if the process of cell lysis is initiated somehow on an MSC by the complement system, then it is almost impossible to stop the killing [90]. 

Multiple external factors have been studied to improve the allogenic survival of MSCs and counteraction against the complement system. Scientists have used anti-C5 antibodies treatment to MSCs before the infusion, as well as the transfection of recombinant adenovirus for the specific overexpression of CD55 inhibitor [90]. Li et al. recognized MSCs propagated antigen in vitro, which is a naturally occurring antibody and has the potential to activate the host complement response. In addition, they demonstrated a simple and economical method to generate heparin-coated MSCs to inhibit the complement system within microenvironments [98]. Besides that, the combined pretreatment of MSCs with IFN-γ and TNF-α has proven to be the best method to inhibit the complement system. This treatment resulted in the secretion of factor H, which is the primary complement inhibitor produced by MSCs [97]. We will discuss this pretreatment application in detail later in this review.

## 3. Therapeutic Role of Modified MSCs

### 3.1. Unveiled the Role of MSCs in Therapeutics

MSCs have become an alluring topic of research due to their role in modern-day therapeutics. Recently, a new therapeutic paradigm has emerged using MSC-derived exosomes and modified MSCs. The exosomes (extracellular vesicles) are rich in lipids, proteins, mRNAs, tRNAs, long non-coding RNAs, micro-RNAs as well as mitochondrial DNA, which is transferred between cells in both near and distant vicinities [99,100]. Research evidence has shown that MSC-derived exosomes exert helpful effects on different disease models such as myocardial infarction [101,102,103,104], hepatic fibrosis [105,106,107] and cancer [108] [107,109,110,111,112]. Additionally, the modified MSCs strive in their effects with the inflammatory factors and the microenvironment of MSCs, which are responsible for their phenotypic effects being exerted on the immune system [113]. 

Inflammation makes MSCs secrete molecules such as IL-10, galectins, IDO, and PGE2, heme oxygenase-1 (HO-1), TNF-inducible gene-6 protein (TSG6), chemokine ligand-2 (CCL2) and NO which are responsible for tissue homeostasis [114]. In addition to that, HLAG-5 secreted by MSCs has the ability to interact with allo-stimulated T cells which assists them to suppress T-lymphocytes, NK cells and regulatory T cells (CD4+CD25highFOXP3+ T cells) [115]. These effects were analyzed by comparing the secretion patterns among molecules of MSCs from murine and humans. Their results showed that IDO and HLA-G are the key effectors from human, whereas NO was from murine MSCs. Also, for inducing the immune response, it was IFN-γ in humans for induction of IDO, but both IFN-γ and TNF-α contributed to iNOS (inducible NO Synthase)/NO induction in murine [29]. 

Chinnadurai et al. studied this immunosuppression by analyzing basal and inducible MSCs secretory molecular patterns, which are responsible for the suppression of T cell proliferation. They identified IDO as a definitive enzyme, which plays a dominant role in MSC-mediated inhibition of T cell proliferation. Furthermore, the blocking of VEGF, granulocyte-colony stimulating factor (GCSF), CXCL9, CXCL10, IFN-α, CCL2 and IL-7 failed to inhibit MSC’s effect on blocking the T cell proliferation. However, this correlation was lost in frozen-thawed MSCs [116]. The potency of MSCs is only possible after some modifications prior to them being used therapeutically. This was shown by Kim and Jang et al. who experimented with IFN-γ primed MSCs to study their influence on IDO activity for progressive inhibition of cell-mediated immunity in graft-versus-host disease (GvHD) [116,117]. 

### 3.2. MSCs Priming and Treatment

Modified or treated MSCs found to be enhanced in their immunomodulatory effects on the immune system. IFN-γ is a key regulatory cytokine due to which MSCs are privileged for immunosuppressive functions [118]. Further modifications of MSCs such as homing at targeted sites can promote not only the escape, but also help in the migration of MSCs towards secondary lymphoid organs. The expression of C-C chemokine receptor type 7 (CCR7) gene after bioengineering has shown enhanced immunomodulation and tolerance [119]. The expression of GCSF, CXCL9, IL-7, and CCL2 by MSCs during the interaction with activated peripheral blood mononuclear cells (PBMCs) explains the mechanism of immunosuppression of MSCs. Some reports also showed that the secretion of VEGF is only possible with fresh MSCs, otherwise, there was a complete loss of VEGF production and T cell suppression activity. Such a case was studied with a comparison between fresh and frozen-thawed MSCs [116].

Previously, glucocorticoids, budesonide or dexamethasone-treated MSCs have shown IFN-γ stimulatory effects. The treatments lead to an enhancement of therapeutic potential of MSCs by inhibiting active inflammatory cytokines and raising tolerance towards GvHD and Crohn’s disease [120,121,122,123,124]. In 2010, Dey et al. found beneficial effects by treating mice with genetically engineered MSCs in cases of Huntington disease. MSCs from brain-derived neurotrophic factor (BDNF) or nerve growth factor (NGF) transgenic mice could create microenvironments in the striatum, which ultimately slowed the neurodegenerative process [125]. In 2013, Kwon showed TNF-α-priming MSCs may manifest the inhibition of tissue necrosis along with the promotion of endothelial progenitor cells homing and angiogenesis in the ischemic hind limb animal models [126]. Recently, Kim et al. showed that IFN-γ-priming of human MSCs resulted in the enhancement of immunosuppressive properties. In contrast, treatment with anti-IFN-γ antibody impairs the properties [117]. Hence, the priming of MSCs with different factors opens a wide spectrum for its therapeutic applicability. A brief update for the sites and sources for mesenchymal stem cells used as a therapeutic tool in various models is given in Table 1.

### 3.3. Outcome of Modified MSCs: Positive and Negative Aspects 

The immunosuppressive and immunomodulatory action potential of MSCs has made them a double-edged sword, which can act favorable as well as against the therapeutics. In 2018, Wang et al. showed that IL-35 gene-modified MSCs exhibited better protective effects on Concanavalin A (Con A)-induced autoimmune hepatitis. IL-35 is required for the regulatory and suppressive functions of Tregs. By a gene-delivery vehicle, IL-35-expressing MSCs decrease IFN-γ and Fas ligand (FasL) levels in mononuclear cells through the Janus kinase 1 (JAK1)-signal transducer and activator of transcription 1 (STAT1)/STAT4 signal pathway and eventually inhibit the hepatocyte apoptosis [142]. 

The behavior of MSCs towards and within its microenvironment is highly complex and needs further research. One of many versatilities was reported by Galland et al., when the group correlated the immunomodulatory effect on NK cells between intra-tumor (T) and adjacent non-tumor tissue (N)-extracted MSCs. The results were astonishing, as they found out that tonsil-derived mesenchymal stem cells (T-MSCs) were more potent immunosuppressive agents as compared to N-MSCs. T-MSCs showed dominance in affecting the NK cells function and phenotype as confirmed by CD56 expression. Upon detailed insight observation, they concluded that tumor-derived MSCs have a definite mechanistic pathway to block the activity of NK cell subsets [145]. The microenvironment of MSCs treatment affects NK cells very deeply and easily. This may be the reason why these cells act favorably depending upon the microenvironment. 

In contradiction with this property, Fregni et al. reported that the tumor microenvironment of MSCs implies some of the selected genes to overexpress and promotes metastasis in the case of lung cancer-derived MSCs. Here, the overexpressed genes were found out to be tumor-initiating markers and progressive towards metastasis [146]. MSCs can be miscreant due to their immune-microenvironment modulatory property, one recent example was the study of MSCs residing in the tumor microenvironment, where they developed therapy resistance in tumor cells [147]. However, these effects were influenced by gemcitabine which made them secrete CXCL10, consequently activating the CXCL10-CXCR3 axis in tumor-initiating cells. Hence, MSCs should not be held responsible for resistance in the chemotherapy of pancreatic adenocarcinomas [147] as the results discussed here are influenced by many factors. Therefore, we believe that MSCs could work against the favorable therapeutics, if not applied with prior extensive research.

## 4. Role of MSCs in Adipocyte Vicinity

It is well known that high-fat diets can induce obesity which represents the risk factors for the development of insulin resistance (IR) and type 2 diabetes (T2DM) [148,149]. Obesity-related diseases such as T2DM induce diabetic wounds and are also associated with rapid cartilage loss and osteoarthritis [150,151,152]. Among the sources of MSCs, adipose tissue is the preferred provider of adipose-derived mesenchymal stem cells (AD-MSCs) [153]. There has been an increase in interest for its therapy potential toward wound healing, tissue engineering and hepatocellular carcinoma [154,155].

### 4.1. Endocrine Function of Adipose Tissue and MSCs Within

Adipose tissue plays an important role in continuing optimal lipid and glucose homeostasis [156]. The adipocytes possess an endocrine system that helps them to alter metabolism known as adipokines [157]. Before going into the details, we would like to shed some light on the role of TGF-β/BMP (bone morphogenic protein) signaling towards the adipogenic and osteogenic differentiation. Both TGFβ and BMPs are recognized to possess dual differentiation function with MSCs and it has also been observed that upon TGF-β/BMP stimulation, the overall expression of runt-related gene 2 (Runx2/Cbfa1) and peroxisome proliferator-activated receptor-γ (PPAR-γ) can be regulated [158]. Recently, studies have shown BMP2 to be forcing MSCs towards both adipogenic or osteogenic differentiation, while TGF-β was found to be inhibiting this development [159,160]. 

**Figure 3:**
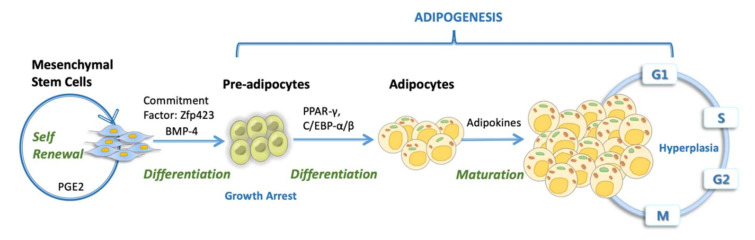
Progression of adipogenic MSCs differentiation and maturation into mature fat cells due to an excess of calories.

In Figure 3, we characterized the important contributions of MSCs in adipocyte vicinity by undergoing the process of adipogenic differentiation. Previously, research revealed that BMP2- and BMP4-expressing cells in cultured fibroblasts are committed towards adipogenesis [161,162,163]. PPAR-γ plays an imperative role in adipocyte differentiation during adipogenesis in vitro [164] as well as in vivo [165,166,167]. During adipogenesis, MSCs start expressing transcription factor ZFP423 (Zinc finger protein 423) for committing to pre-adipocyte lineage and the produce regulators PPAR-γ and C/EBP-α/β (transcription co-activators CCAAT/enhancer-binding protein α and β) for differentiation and maturation [168,169,170]. Hence, MSCs residing in the adipose tissue contribute towards healthy mass storage by their differentiation into adipocytes. 

### 4.2. Loss of Potency of MSCs

**Figure 4:**
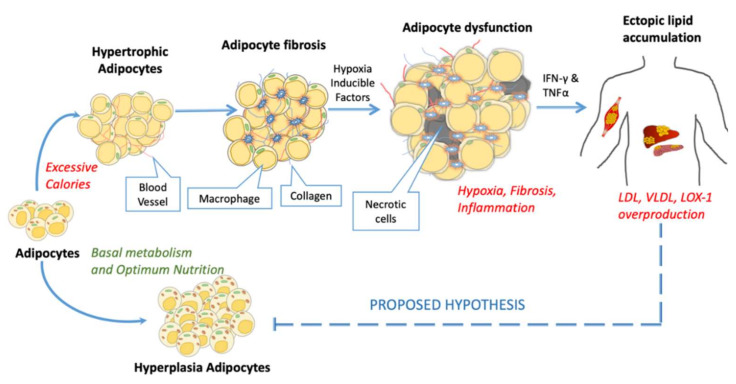
The above figure demonstrates the failure of overnutrition accommodation resulting in adipose tissue dysfunction. Insufficient pre-adipocytes pressure the matured adipose cells to undergo hypertrophy resulting in adipocyte fibrosis (macrophage accumulation and collagen deposition). Furthermore, these cells progress towards adipose dysfunction (ectopic lipid deposition leads to hypoxia and necrosis).

There are suggestions from many teams that dysfunctional adipocytes in obese or diabetic patients are due to pro-inflammatory cytokines [171,172,173,174]. In our previous work, understanding the mechanism of electronegative low-density lipoprotein (L5 LDL) involved adipose tissue inflammation; we observed that atherogenic lipid deposition and excessive hypertrophy resulted in macrophage infiltration and adipose tissue dysfunction [175]. Figure 4 highlights the hallmarks of adipose tissue dysfunction. In the case of overnutrition, the storage of extra calories in adipose tissue is needed, and to accommodate extra calories, either adipose tissue needs to expand (hypertrophy) or increase its number (hyperplasia). The excessive fat overload leads to an increase in serum LDL and VLDL, which further promotes atherogenic lectin-like oxidized low-density lipoprotein receptor-1 (LOX-1) overexpression, these interactions result in variable postliminary processes depending upon microenvironment conditions and type of cells [176]. The proposed hypothesis in Figure 4 suggests the possible links of a feedback loop mechanism of adipocyte dysfunction which promotes adipocyte hypertrophy and dysfunction by inhibiting hyperplasia.

Adipocyte hyperplasia or hypertrophy occurs in an operative state by a distinct secretory pattern of adipokines of these cells [177,178]. An impaired multipotency of MSCs was found in T2DM cases, where oxidative stress impaired the blood flux recovery [179]. Adipogenic MSCs from the diabetic patients failed to differentiate into fully functional adipocytes. Hence, insulin resistance might advance via hypertrophy of existing mature adipocytes [180]. The mechanistic view behind this impairment is hyperinsulinemia induced Nox4 (NADPH oxidase 4) triggering oxidative stress leading to restrict multipotency and increases adipogenic predictions of diabetic mice [181]. Additionally, insulin treatment of WT-MSCs elevated the expression of Nox4 and ultimately increased the rate of differentiation into adipocytes [181,182,183]. 

### 4.3. Need for Therapeutic Targets 

Yan et al. unveiled that MSCs derived from T2DM are compromised in their multipotency which made them boost post-ischemic neovascularization in diabetic mice [181]. In 2016, Zoelen et al. showed that TGF-β promotes osteogenic differentiation of hMSCs while at the same time inhibiting adipogenic differentiation by lowering the expression of PPARγ, ADAMTS5, and AKR1B10. They concluded that these findings might support the therapeutic capabilities for preventing osteoporosis and obesity [160]. However, antidiabetic TZDs to target PPARγ, exaggerate the expression of oxidized LDL receptor 1 (OLR1) in adipocytes. Hence, targeting PPARγ alone may worsen the obesity seen in other metabolic diseases [156]. The mechanism of fat mass expansion is poorly understood. Earlier, scientists concluded that adipocyte hypertrophy comes down to fat mass expansion [184,185,186] and they justified that this condition is due to a fixed number of pre-adipocytes in adults [187,188,189,190]. Later, scientists proved this by tailoring the isotopic methodology to track down the process of adipogenesis with rare stable isotopes. Some groups were successful in identifying the adipocyte hypertrophy as the dominant mechanism of adult fat mass expansion by using mass spectrometry [191,192].

## 5. MSCs Response and Potential Towards Diabetes

Diabetes affect millions of people and is considered as a worldwide epidemic [193]. T2DM covers 85-95% of overall diabetes cases, having insulin resistance or problem with the insulin secretion by pancreatic β-cells [194]. MSCs have served to generate insulin-secretory cells, increase in islet engraftment and survival, and also be useful in treating diabetic ulcers and limb ischemia [195,196,197,198]. They also facilitate a micro-environmental niche by the secretion of some paracrine factors and deposition of extracellular matrix [51,199,200,201,202,203]. Therefore, MSCs have huge potential for diabetic therapeutics.

### 5.1. Direct therapeutic use of MSCs

There are many reports on animal models for dose-dependent therapeutics of MSCs infusion (mixed MSCs from different sites). Due to a lack of standardization, there are great variations within clinical trials regarding MSCs dosage and the frequency [120,121,122,204,205,206]. However, the factors influencing MSCs therapy are the type of MSCs (isolation and source dependent), administration of delivery, viability and purity of MSCs. Besides these factors, the most important issues are the stages, types and conditions of diabetic patients [207]. This was supported by a study in which researchers administered a direct injection of the MSCs for both the single as well as multiple times to diabetic rats that improved hyperglycemia in 4 weeks [200]. Similarly, in some other studies for the treatment of diabetic patients, scientists considered a single injection to not be enough, therefore, they administered mainly 2-4 times a day for 2 to 12- weeks, multiple injections which resulted in improvement of the patients [208,209,210,211]. In 2014, Bhansali et al. demonstrated 9 out of 11 diabetic patients reached the endpoint, and insulin requirement was lowered down by 66.7%. Also, the same group found that 7 out of 10 patients ended with 75% lower insulin requirements. Additionally, three out of them were able to discontinue the insulin completely after a single BM-MSC autologous transplantation. However, they did not mention the effective duration in their reports [208,212]. These reports still need some clarification regarding how they understood the mechanism of improvement in diabetes through MSCs therapeutics (for quality assurance). 

### 5.2. MSCs and Their Exertion in Diabetes 

It was Chen et al. who first made the incompletely differentiated MSCs into insulin-producing cells (IPCs) derived from rat, which successfully expressed insulin and nestin [213]. Later, MSCs were found to be successfully promoted islet of beta cells even after hypoxia and oxidative stress [214,215]. Until now, more than 96 phase I/II clinical trials have been attempted for the treatment of diabetes (https://clinicaltrials.gov/ct2/results?cond=Diabetes+Mellitus&term=MSC&cntry=&state=&city=&dist=), yet only few of them (Table 2) were considered promising for the effect of MSC treatment in the management of T2DM [216].

#### 5.2.1. MSCs Clinical Trials and Combination Therapy

The randomized trials of phase 1 were first studied by a combined infusion therapy. Bone marrow derived mononuclear cells (BM-MNCs) and hyperbaric oxygen treatment combination demonstrated that infusion formula between two or more is beneficial, although both failed to synergize [217,218]. The most important scrutiny in clinical trials is the ratio of patient safety to risk. In that concern, there must be an evaluation of MSCs treatment in T2DM cases for analysis of detrimental events. In many studies, no acute and immunological events were noted, as the hypothetical risks involved here, pulmonary and upper respiratory destructions by intravenous injection which may be followed for interspersing cell fleeting through lungs, bruises caused by perforation, all these studies noticed no such developments [208,209,210,212,217,218,219,220,221,222,223,224,225]. However, mild nausea, headache, vomiting and abdominal pain were noted after MSC transplantation [212,219,222]. Therefore, the response of MSC towards diabetes is very optimistic and can be considered as a therapeutic option.

Bhansali et al. performed an infused transplant of autologous BM-MSCs and BM-MNCs in T2DM cases and found improvement in insulin sensitivity in 67% of patients. Besides this, they also concluded that MSCs mediated the IRS-1 gene expression for the betterment of insulin sensitivity, while MNCs boosted the C-peptide response [226]. Similar infusion results were obtained by Wang et al. [224]. However, the withdrawal technique of BM-MSCs and BM-MNCs from the femur or iliac crest is painful and could cause infection [227]. 

The increase in C-peptide and decrease in HbA1c are considered to be two major positive outcomes of MSC therapy. Guan et al. explored such improvements with WJ-MSCs therapy treatment twice in diabetes patients [228]. Taking a message from these results, the number of T-reg cells was also increased after infusion of WJ-MSCs with UC-MSCs, followed by reduced insulin dose [210]. Besides these trials, the motive of stem cell therapy was to reverse the insulin resistance and improvement in immune dysfunction. To some extent Zhao et al. found some positive results of reversing the immune dysfunction. They observed metabolic improvement and balancing between Th1/Th2/Th3 cytokine secretion using CB-MSCs, but the reversal in insulin resistance was not achieved [225].

#### 5.2.2. Mechanistic Details of MSCs with Clinical Trials

Along with immunomodulatory and immunosuppressive roles, the regenerating capability of MSCs make them unique and a very suitable candidate for cell-based therapy in autoimmune and inflammatory disorders [229,230,231]. The differentiation potential of MSCs into IPCs is the most striking of their features, which can be used to ameliorate hyperglycemia. The differentiation of endocrine portions within the pancreas is strictly regulated by transcription factors Pdx-1, Ngn-3, NeuroD1, Pax4 and Pax6 [232]. For the equitable reprogramming of these cells, it becomes mandatory for the MSCs to differentiate into IPCs. In 2005, human BM-MSCs were successfully differentiated into IPCs with the use of adenoviral vectors encoding for murine Pdx-1. Later, Xie et al. studied the differentiation of human BM-MSCs into IPCs by a three-step process and resulted in them becoming insulin-secreting cells in a glucose dose-dependent manner [233,234]. Similarly, Nam et al. performed in vitro differentiation of human eyelid AD-MSCs into IPCs and transplanted them into T2DM mice model [235]. Out of the two groups; T2DM and control, T2DM mice group experienced IPCs mediated improvement, high IL-6 and also an increase in circulating insulin level promoting the metabolic improvements.

### 5.3. Possible Approaches of MSCs prior to Clinical Trials 

Functions of stem cells are highly dependent on the microenvironment, which is strictly regulated by its ECM (extracellular matrix), growth factors and immune cells [236]. ECM of MSCs has a great potential for the quality improvement of MSCs such as adhesion and proliferation. Disturbed metabolic conditions and hyperglycemia often leads to organ damage and also hampers the quality of MSCs, which is a major hurdle of autologous clinical application. Therefore, we highly recommend a particular kind of 3D (three dimensional) ECM culture, which may be used to enhance such functions of MSCs [237]. For instance, Block et al. used 3D-ECM culture for a phenotype of interest on the basis of cell size and stage-specific embryonic antigen-4 levels [238]. These kinds of findings may assure the autologous use of MSCs, with high quality and genetic modifications in order to improve the therapeutic potential of MSCs in several diseases and disorders [239]. 

The survival rate of MSCs is an essential quality factor for clinical trials. We have discussed the loss of potency in frozen-thawed MSCs [240], but in an experiment of allergic asthma, frozen-thawed MSCs were as efficient as the fresh MSCs [241]. Hence, we need more light to explore the features of frozen-thawed MSCs as compared to fresh MSCs. Apart from that, some scientists have explored the challenges of MSCs to sublethal risks of cellular stress in vitro, i.e., hypoxia, heat or compromised nutrition. With preconditioning incubation, MSCs demonstrated a reduction of cell apoptosis in vivo while maintaining their biological functions [242,243]. When comparing hypoxia and normoxia exposure to cells *in vitro*, hypoxia-treated MSCs emerged to show an increase in population multiplying rate [244,245]. This phenomenon can be explained by understanding the role of stabilizing factor HIF-1α. During hypoxia, the expression of HIF-1α rises by 3.4 fold in MSCs that in turn to reduce the ROS (reactive oxygen species), block the oxidative phosphorylation and promote glycolysis [242,245]. Besides that, HIF-1α also activates NF-κB to downregulate the level of Bcl (B cell lymphoma) and caspase 3, and activation of PrPc (cellular prion protein) enhances the superoxide dismutase (SOD) and catalase to attain complete protection from oxidative stress [242,246].

Enduring oxidative stress in hypertension and diabetic conditions, cells are influenced by ROS [247]. High-calorie diet induces ROS accumulation in the adipose tissue of the T2DM mice model and promotes senescence of MSCs by expressing β-galactosidase and p53 [248]. Besides, the viability of MSCs is severely hampered by oxidative stress [249,250]. Thus, the direct use of MSCs for treatment may not be practical. In 2016, Cheng et al. observed the upregulation of *sex-determining region Y-box 2*, (*SOX2*), *Oct-4*, and *NANOG* in high glucose-treated MSCs by the influence of intracellular ROS formation, signifying the improvement in the stemness of MSCs [251]. These pieces of evidence show preconditioning of MSCs may be of help in maintaining function and enhancing survival rates in clinical trials.

## 6. Perspective

MSC therapy exemplifies a materializing style of modern alternative treatment with the retention to hold site-specific immune regulation that controls T cells in autoimmune diseases and allograft rejection. The current review article on immunosuppressive properties holds great confidence for treating immune-mediated diseases, obesity, CVD and diabetes. However, it is also mandatory not to augment the therapeutic potential as many unanswered questions need elucidation before making promises. MSCs are known to bear effects in vascular repair by enactments into blood vessels, differentiation into endothelial cells, pericytes and other vascular phenotypes [252,253,254], which is further supported by autocrine and paracrine properties by producing vascular growth factors and proangiogenic cytokines [255,256]. As far as the endocrine properties of MSCs are concerned, it is credible that the classification of admissible effector molecules could lead to novel treatment and rehabilitation of cellular therapy with MSCs [29].

There is very limiting but reassuring data available for ameliorating glycemic controlled MSCs therapy in T2DM cases. We have highlighted many experiments to overcome hyperglycemia such as differentiating MSCs into IPCs (insulin-producing cells), mitigating insulin resistance, conversion of alpha cells to beta cells and remodeling pancreatic regeneration. However, animal models used for the aforementioned analysis did not equal human T2DM patients and hence, the underlying scheme involved here must be cross-examined in detail.

Coordinators for upcoming research must incorporate a definite identification of cell markers such as the marker explored by Hudak et al. [257]. Standardized and validated isolation and culture protocols with lineage differentiation and stimulation methods, which may ease the animal and clinical studies. Additionally, cell modification, injection frequency and dosages are required to be studied in detail to further guide the therapeutic potential of MSCs. An exceptional insight of this compelling cell population might be apprehensive of a contemporary therapeutic scheme to recover the immune response in an array of immune-mediated diseases, obesity, CVD and diabetes.

## Figures and Tables

**Table 1:** Therapeutic potential of MSCs tested.

Site of Isolation	Subject tested	Role of MSCs	Modifications	Applications	Therapeutic Potential	Ref.
BM-MSC	Rat	Injury healing	_	Autologous mode	Traumatic brain injury	[127]
BM-MSC	Mouse	Injury healing	_	Allogenic mode	Traumatic brain injury	[128,129]
AD-MSC	Human	Knee injury healing	_	Autologous	As a treatment for osteoarthritis	[130]
BM, AD, UC and WJ-MSC	Human	Immunosuppression	IFN-γ treated MSCs	Allogenic and autologous mode treatment and Prevention	GvHD	[131]
BM-MSC	Human	Immunosuppression	_	Autologous	Multiple sclerosis	[132,133]
AD-MSC BM-MSC	Mouse	Antimicrobial and anti-infection	Collagenase 0.1% treatment	Allogenic and in vitro mode	Cystic fibrosis	[134]
UC-MSC	Mouse human	Immunosuppression	Vehicle-controlled without modification	Autologous and allogenic mode	Cirrhosis and autoimmune diseases	[135]
UC, AD, BM-MSC, Placenta	Human	Anti-inflammation, immunosuppression	_	Exogenous MSCs	Bronchopulmonary dysplasia	[136]
BM-MSC	Human	Immunomodulation	_	Autologous and Allogenic	Acute myocardial infarction, chronic ischemic heart disease, cardiomyopathy	[137]
BM-MSC	Mouse	Anti-inflammation, immunosuppression	Marrow-derived clonal MSC	Autologous	Inflammatory bowel disease	[138]
UC-MSC	Human	Immunosuppression	_	Allogenic	Systemic lupus erythematosus	[133]
BM-MSC	Mouse	Anti-inflammation	_	Allogenic	Chronic wound healing	[139]
BM-MSC	Human	Immunosuppression, immunomodulation	_	Autologous	Drug-resistant epilepsy	[140]
BM-MSC	_	MSCs cloning and proliferation	Feta bovine serum	Only Cultured	Chronic heart failure	[141]
AD-MSC	Mouse	Immunosuppression, immunomodulation	IL-35 gene modified	Exogenous	Con A-induced liver injury	[142]
BM-MSC (Purchased)	In *vitro*	Immunosuppression	Intracellular delivery of steroids	Allogenic Co-cultured	GvHD, Crohn’s disease	[124]
BM-MSC	YAC128 Mouse	Anti-inflammation, immunosuppression	Over-expressing *BDNF* and *NGF* genes	Autologous to mice models	Huntington’s disease	[125,143]
BM-MSC	Rat	Anti-inflammation, immunomodulation	Long term clonal MSCs	Reduces Fibrotic scars	Rat spinal cord injury	[144]

BM-MSC: bone marrow-derived mesenchymal stem cell; AD-MSC: adipose tissue-derived MSC; UC-MSC: umbilical cord-derived mesenchymal stem cell; WJ-MSC: Wharton’s Jelly-derived MSC; IFN-γ: interferon-γ BDNF, brain-derived neurotrophic factors; NGF, Nerve growth factor; GvHD: graft versus host disease; Con A: concanavalin A.

**Table 2:** Details of the clinical trials using MSCs on diabetes mellitus.

Status	Outcomes/Complications		Criteria		NP	Treatment approach	Mode of intervention	Center/NCT No.
	Primary	Secondary	Inclusions	Exclusions				
Completed	Reduction (≥50%) of insulin dose	HbA1c increases	T2DM for 5 years, 3 months’ medication before therapy, HbA1c range: 7.5% to 9%	Type 1 diabetes, chronic or severe diseases	30	2 treatment at 6 months of interval	BM-MSCs, autologous inoculation	VRISCGT Hanoi Vietnam, *NCT03343782*
Unknown; crossed the completion date	Reduction of insulin dose, change of C-peptide levels vs. baseline	Evaluation of adverse events e.g., fever, allergy	T2DM of age 18-80, ITT indicating insulin resistance, no infection	Chronic or severe diseases, HIV, Hepatitis B or C infection	30	2 treatment at 3 months of interval	UC-MSCs, allograft and intravenous	Shandong University, China *NCT01413035*
Completed	NF-κB inflammatory markers and osteoblast-specific gene expression	NF-κB inflammatory markers and apoptotic marker	T2DM of age 18 and above, HbA1c range between 6.5%	Receiving TZD, steroid or other medication, high serum creatinine 1.4 mg/dL for female and 1.5 for male	75	Cross-sectional 2-4 weeks of time frame	Not mentioned	Chiang Mai University, Thailand *NCT02286128*
Unknown; crossed the completion date	Reduction of insulin dose, HbA1c increases	Adverse events	T2DM of age 18-75, ITT indicating insulin resistance	Severe diseases, HIV, Hepatitis B or C infection, pregnant	24	One-year time frame, 0 to 14 ±2 days 3 times	BM-MSCs intravenous	APGH, Beijing China, *NCT01142050*
Completed	HbA1c monitored for 1 year, results not posted	Insulin dose, severity of adverse events	T2DM of age 35 or above, HbA1c range between 7.5% to 12%	Insulin requirement above 100 U/day, proteinuria, chronic or severe diseases receiving TZD, steroid or other medication	22	Cross-sectional for 1 year; Number of times not specified	Autologous BM-MSCs infused with BM-MNCs with insulin drug	FGH, Fuzhou China, *NCT01719640*
Unknown; crossed the completion date	HbA1c monitored for 1 year	Fasting blood glucose monitored for 1 year	T2DM of age 35 to 65, HbA1c range between 7.5% to 11%	Insulin requirement above 100 U/day, proteinuria, chronic or severe diseases receiving TZD, steroid or other medication	100	Cross-sectional for 1 year; Number of times not specified	UC-MSCs infused with GLP-1 (Liraglutide)	Diabetes care center of Nanjing Military Command, Fuzhou China, *NCT01954147*
Completed	Reduction (≥50%) of insulin dose	N.A.	T2DM of age 30 to 70, HbA1c below 7.5%	Type 1 diabetes, severe diseases, HIV, Hepatitis B or C infection	30	6 months cross-sectional	Autologous BM-MSCs infused with vitamin B and MNCs	PIMER Chandigarh, India, *NCT01759823*
Unknown; crossed the completion date	Osteoporosis in T2DM patients	N.A.	T2DM patients of age 40 to 99	Organization people	1200	3 years cross-sectional	Not provided	NTUH, Taipei, Taiwan, NCT*01846533*
Active but not recruiting	CTCAE-assessment of 12 months and change in hypoglycemia	Fasting glucose monitored for 1 year, change of C-peptide and HbA1c	Type 1 diabetes detection less than 6 weeks, antibodies against pancreatic β-cells	Pregnant or breastfeeding, cancer or severe diseases, known HIV, Hepatitis B or C infection	20	12 month cross-sectional, time frame of weeks 0 and 3	Intravenous injection of autologous BM-MSCs	RIT, Tehran Iran, *NCT04078308*
Completed	Change of C-peptide	N.A.	Type 1 diabetes detection within 10 days, fasting C-peptide below 0.12nmol/L	BMI, immuno-suppressive treatment, HIV, Hepatitis B or C infection, pregnant	20	1-year follow-up study	Intravenous injection of autologous BM-MSCs	UUH, Uppsala, Sweden *NCT01068951*
Terminated	Change of C-peptide	N.A.	Type 1 diabetes detection within 3 weeks, fasting C-peptide below 0.12nmol/L	BMI, immuno-suppressive treatment, HIV, Hepatitis B or C infection, pregnant	50	2-year follow-up study	Intravenous injection of autologous BM-MSCs	UUH, Uppsala, Sweden *NCT02057211*
Unknown; crossed the completion date	Change of C-peptide OGTT curve	Fasting blood glucose monitored for 1 year, a decrease in HbA1c	Type 1 diabetes of age 35 or below, HbA1c 7.5% or above	Insulin requirement above 100 U/day, proteinuria, chronic or severe diseases, receiving TZD, steroid or other medication	44	1-year follow-up study	Intravenous injection of allograft UC –MSCs infused with pancreatic MNCs	FGH, Fuzhou China, *NCT01374854*
Ongoing	Change of C-peptide	Change of C-peptide and change in β-cell function	Type 1 diabetes detection within 3 months, male and female of age 12 and 30 years	Body Mass Index < 14 or >35, HbA1c >12%, and/or fasting blood glucose >270 mg/d	50	1-year follow-up study	Allograft BM–MSCs infused with plasmalyte 0.5%	MUSC, South Carolina USA *NCT04061746*
Ongoing	Safe assessment of Allogenic use	N.A.	Type 1 diabetes of age 18 to 35, HbA1c change, baseline C-peptide	Type 1 diabetes below 18 and above 35 age, pregnant or comatose	20	1-year follow-up study, 2 dosages at the interval of 6 months	Allogenic AD-MSCs infused with BM-MNCs intravenously	Cell Therapy Center, Amman, Jordan, *NCT02940418*
Enrolling by invitation	Pancreatic β-cells monitoring by follow-up for 2 years and change of C-peptide analysis	Oral cholecalciferol 2000UI/day supplementation for 2 years	Type 1 diabetes detection within 4 months and pancreatic autoimmunity	HIV, Hepatitis B or C infection, pregnant, cancer	30	1-year follow-up study	Allogenic AD-MSCs along with oral cholecalciferol supplementation and vitamin D	CFFUH Rio De Janeiro, Brazil, *NCT03920397*

NP: Number of participants; N.A.: Not Available; NF-κB: Nuclear factor-κB; VRISCGT: Vinmec Research Institute of Stem Cell and Gene Technology; ITT: Intravenous insulin tolerance test; APGH: Armed police general hospital; FGH: Fuzhou general hospital; PIMER: Postgraduate Institute of Medical Education and Research; NTUH: National Taiwan University Hospital; BM-MNCs: Bone marrow mononuclear cells; CTCAE: Common terminology criteria for adverse events; RIT: Royal Institute of Tehran; UUH: Uppsala University Hospital; MUSC: Medical University of South Carolina; CFFUH: Clementino Fraga Filho University Hospital; NCT number refer to its www.clinicaltrials.gov identifier.

## Abbreviations

ADAMTS5	A disintegrin and metalloproteinase with thrombospondin motifs 5
AKR1B10	Aldo-keto reductase Family 1 Member B10
Akt-MSC	Protein kinase B recombinant MSCs
APCs	Antigen presenting cells
BCL	B Cell Lymphoma
BDNF	Brain-derived neurotropic factor
BMP	Bone morphogenic protein
C/EBP	CCAAT-enhancer binding proteins
Cbfa1	Core-binding factor alpha 1
CCL2	Chemokine ligand-2
CD	Cluster of differentiation
C-MSC	Cord tissue derived MSCs
COX-2	Cyclooxygenase 2
CR3	Complement Receptor 3
CVD	Cardiovascular disease
CXCL	Chemokine Ligand
CXCR	Chemokine receptor
ECM	Extracellular Matrix
Foxp3+	Fork-head box P3
GCSF	Granulocyte-colony stimulating factor
GDM	Gestational diabetes mellitus
HGF	Hepatocyte growth factor
HIF-1α	Hypoxia inducible factor-1α
HLA-G	Human leukocyte antigen-G
HO	Heme-oxygenase
IBMX	3-isobutyl-1-methylxanthine
IDO	Indolamine 2:3-dioxygenase
IL	Interleukin
IR	Insulin resistance
IPC	Insulin producing cell
iNOS	Inducible NO synthase
MAC	Membrane attack complex
MMP-9	Metalloproteinase 9
MIP α/β	Macrophage inflammatory protein
MNC	Mononuclear cell
MSC	Mesenchymal stem cell
N-MSC	Non-tumor MSCs
Ngn 3	Neurogenin 3
NO	Nitric oxide
Nox4	NADPH oxidase 4
04-Oct	Octamer-binding transcription factor 4
OLR1	Oxidized LDL receptor 1
P-MSC	Placenta tissue derived MSCs
Pax 4	Pairedbox 4
PBMC	Peripheral blood mononuclear cell
Pdx-1	Pancreatic and duodenal homeobox-1
PGE2	Prostaglandin E2
PGH2	Prostaglandin H2
PPAR-γ	Peroxisome proliferator-activated receptor-γ
RANTES	Regulated on activation, Normal T cell expressed and secreted
ROS	Reactive oxygen species
Runx2	Runt-related gene 2
SOD	Superoxide dismutase
SOX2	Sex-determining region Y-box 2, (SOX2)
T1DM	Type 1 diabetes mellitus
T2DM	Type 2 diabetes mellitus
TB4	Thymosin β-4
TNF-α	Tissue necrosis factor-α
Treg	Immunoregulatory T cell
T-MSC	Tumor derived MSCs
TGF-β	Transforming growth factor-β
TSG6	TNF-inducible gene -6 protein
TZD	Thiazolidinedione
VEGF	Vascular endothelial growth factors
ULBP	UL16 binding protein
ZFP423	Zinc finger protein 423
